# Whole Prostate Volume and Shape Changes with the Use of an Inflatable and Flexible Endorectal Coil

**DOI:** 10.1155/2014/903747

**Published:** 2014-10-13

**Authors:** Murat Osman, Haytham Shebel, Sandeep Sankineni, Marcelino L. Bernardo, Dagane Daar, Bradford J. Wood, Peter A. Pinto, Peter L. Choyke, Baris Turkbey, Harsh K. Agarwal

**Affiliations:** ^1^Molecular Imaging Program, NCI, NIH, Bethesda, MD 20892, USA; ^2^George Washington University, Washington, DC 20052, USA; ^3^Department of Radiology, Nephrology Center, Mansoura University, Mansoura 35516, Egypt; ^4^Leidos Biomedical Research, Inc., Frederick National Laboratory for Cancer Research, Frederick, MD 21702, USA; ^5^Center for Interventional Oncology, NIH, Bethesda, MD 20892, USA; ^6^Urologic Oncology Branch, NCI, NIH, Bethesda, MD 20892, USA; ^7^Philips Research North America, Briarcliff Manor, NY 10510, USA

## Abstract

*Purpose.* To determine to what extent an inflatable endorectal coil (ERC) affects whole prostate (WP) volume and shape during prostate MRI.* Materials and Methods.* 79 consecutive patients underwent T2W MRI at 3T first with a 6-channel surface coil and then with the combination of a 16-channel surface coil and ERC in the same imaging session. WP volume was assessed by manually contouring the prostate in each T2W axial slice. PSA density was also calculated. The maximum anterior-posterior (AP), left-right (LR), and craniocaudal (CC) prostate dimensions were measured. Changes in WP prostate volume, PSA density, and prostate dimensions were then evaluated.* Results.* In 79 patients, use of an ERC yielded no significant change in whole prostate volume (0.6 ± 5.7%, *P* = 0.270) and PSA density (−0.2 ± 5.6%, *P* = 0.768). However, use of an ERC significantly decreased the AP dimension of the prostate by −8.6 ± 7.8% (*P* < 0.001), increased LR dimension by 4.5 ± 5.8% (*P* < 0.001), and increased the CC dimension by 8.8 ± 6.9% (*P* < 0.001).* Conclusion.* Use of an ERC in prostate MRI results in the shape deformation of the prostate gland with no significant change in the volume of the prostate measured on T2W MRI. Therefore, WP volumes calculated on ERC MRI can be reliably used in clinical workflow.

## 1. Introduction

Prostate volume is an important parameter in prostate cancer screening and in planning radiation therapy [1–4]. Prostate volumes alone are used to predict BPH-related outcomes such as acute urinary retention (AUR) and BPH progression [5, 6]. By normalizing the prostate-specific antigen (PSA) to prostate volume, prostate density has a higher positive predictive value for prostate cancer compared with PSA alone [7, 8]. Moreover, PSA density provides enhanced information on the probability of aggressive prostate cancer versus BPH and the likelihood of biochemical recurrence after treatment [2, 9]. A PSA density threshold of 0.15 ng/mL/cm^3^ is used to decide whether prostate cancer patients are eligible for active surveillance [10, 11]. Additionally, prostate volume measurements are used during radiation therapy planning [12]. Varying prostate volumes have also been seen to affect the accuracy of targeted prostate biopsies [13, 14].

Prostate volumes are traditionally obtained using the ellipsoid formula based on triplanar linear measurements on transrectal ultrasonography (TRUS) [15]. The prostate volumes obtained from TRUS are subject to interreader variability, especially in large and/or irregular prostates, primarily due to the difficulty in delineating apical and basal prostate boundary [16]. The ellipsoid model has been shown to be less accurate than planimetric methods since it assumes that the prostate gland has a normal ellipsoid shape [17, 18]. However, newer automated and semiautomated techniques for accurate prostate segmentation on T2 weighted magnetic resonance images (MRI) are now available and enable more accurate determination of prostate volume [19–21]. The addition of an endorectal coil (ERC) for data acquisition along with phased array surface coils improves the signal-to-noise ratio (SNR) and, thus, the localization and depiction of the prostate gland [22–25]. Recently, it has been reported that the introduction of an ERC changes the prostate shape and decreases whole and zonal prostate volume measurements [26, 27]. However, the prostate gland is not expected to change its volume due to its glandular structure [28]. Therefore the prostate volume should stay constant even while the shape might change [28–30].

In this paper, we assessed the changes in prostate volume and shape with and without ERC during the same scanning session. Based on this data, PSA density was also compared with and without an ERC.

## 2. Materials and Methods

### 2.1. Study Design and Population

In this prospective, single-institution study was approved by the local institutional review board. The study was compliant with the Health Insurance Portability and Accountability Act and informed consent was obtained from each patient. This study included 79 consecutive patients who underwent multiparametric 3T MRI from March 2010 to September 2010 first with surface coil only and then with endorectal and surface coil MRI at 3T in the same imaging session. These patients had a mean age of 64.7 years (median 59.6, range 38–82 years) and a median serum PSA of 7.73 ng/mL (range 1.09–65.20 ng/mL). No patient had undergone prior treatment (hormonal therapy, surgery, or radiation therapy) before MR imaging.

### 2.2. Multiparametric MRI

All MR images were obtained on a 3T clinical MRI scanner (Achieva-TX, Philips Healthcare, Best, The Netherlands). Each patient was first scanned with only the 6-channel SENSE cardiac coil (Philips Healthcare, Best, The Netherlands), henceforth referred to as non-ERC MRI. The non-ERC MR imaging protocol included triplanar T2 weighted (T2W) turbo spin echo (TSE) and axial diffusion weighted imaging (DWI) MRI. Following the non-ERC MRI, an ERC MRI was performed using a combination of an endorectal coil (BPX-30, Medrad Inc., Pittsburgh, PA, USA) and the anterior half of the 32-channel cardiac coil (InVivo Corp., Gainesville, FL, USA) without prior bowel preparation. The ERC was placed using a semianesthetic gel (Lidocaine, AstraZeneca, USA) while the patient was in the left lateral decubitus position. The balloon surrounding the coil was distended with 45 mL of perfluorocarbon (3 mol/L-Fluorinert, 3 M, St. Paul, MN, USA) to reduce susceptibility artifacts induced by air in the balloon. The ERC MR imaging protocol included triplanar T2W TSE, axial DWI, 3D MR Spectroscopy imaging (MRSI), axial precontrast T1W, and axial 3D fast field echo dynamic contrast-enhanced (DCE) MRI. For the purposes of this study, changes in WP volume and shape change were assessed solely on axial T2W MRI. Geometry (slice center and angulation) of the axial slices were defined with respect to the prostate, so that axial slices are orthogonal to the urethra in the mid prostate level for both non-ERC and ERC MRI. Imaging parameters for the acquisition of axial T2W MRI in non-ERC and ERC MRI are summarized in Table 1.

### 2.3. Prostate Volume and Shape Measurements

**Figure 1:**
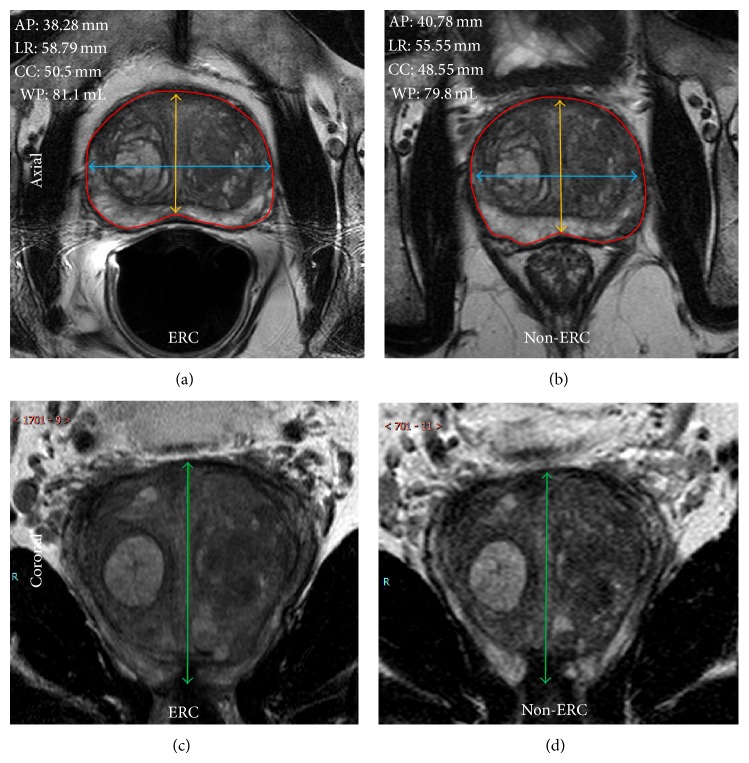
Axial ERC and non-ERC T2W MRI depicting the maximum AP (yellow) and LR (blue) prostate dimensions and WP contours (red) used for volume determination are shown below. Coronal ERC and non-ERC T2W MRI of the same patient were used to measure maximum CC (green) prostate dimension. AP, LR, and CC measurements are also annotated.

A body radiologist with 8 years of experience in MRI used in-house research software to compute the whole prostate volume. The prostate boundaries were contoured on each slice of the axial T2W MRI without any guidance from other MR images (Figure 1). However, the prostate boundaries at the apical and basal level are often not well defined. Therefore, non-ERC and ERC contours were drawn simultaneously to ensure the same structures were identified and contoured on non-ERC and ERC MRI images. Once the whole prostate gland was contoured, the same software was used to determine the whole prostate volume in mL (cm^3^). PSA density was computed by dividing the PSA value at the time of MR exam by the prostate volume.

Prostate shape was evaluated using MIPAV (Medical Image Processing, Analysis, and Visualization, CIT, NIH, Bethesda, MD). For shape evaluation, one T2W MRI slice in the mid axial plane was used to measure maximum prostate size in the anterior-posterior (AP) and left-right (LR) dimensions (Figure 1). Intraprostatic landmarks such as the urethra, BPH nodules, and cysts were used to ensure that these measurements were done at the same prostate level for both non-ERC and ERC MRI in each patient. Maximum prostate CC dimension was measured on coronal T2W MRI.

### 2.4. Statistical Analysis

Wilcoxon's signed rank test was used to compare change in the prostate volume between non-ERC and ERC MRI. A two-tailed paired Student's *t* test was used to compare changes in PSA density and maximum AP, LR, and CC prostate dimension between non-ERC and ERC MRI. *P*-values less than 0.05 were used for statistical significance.

## 3. Results

All 79 patients were successfully scanned with non-ERC and ERC MRI in the same scanning session. The mean, standard deviation, median and range of WP volume, PSA density, maximum AP, LR and CC prostate dimension measured on non-ERC and ERC-MRI are shown in Table 2. The difference (ERC-non-ERC) and percentage difference relative to non-ERC MRI values are also shown in Table 2. The mean whole prostate (WP) volume between non-ERC (57.5 ± 28.4 mL) and ERC MRI (57.9 ± 29.2 mL) was not statistically significantly different (*P* = 0.270). PSA density calculated from volumes obtained with ERC (0.246 ± 0.583 ng/mL/cm^3^) and without ERC (0.245 ± 0.563 ng/mL/cm^3^) were not statistically significant (*P* = 0.768). Out of 79 patients, PSA density of 2 patients increased and crossed the 0.15 ng/mL/cm^3^ PSA density level using ERC. The maximum AP dimension significantly decreased from 34.4 ± 8.0 mm to 31.5 ± 8.2 mm with *P* < 0.001. Similarly, the maximum LR dimension increased from 48.9 ± 6.8 mm to 51.0 ± 6.8 mm with *P* < 0.001, and CC dimension increased significantly from 39.6 ± 8.5 mm to 42.8 ± 8.3 mm with *P* < 0.001 after the use of an ERC. Figure 1 shows the axial and coronal T2W MRI of one patient depicting the change in prostate shape with the use of an ERC.

**Figure 2:**
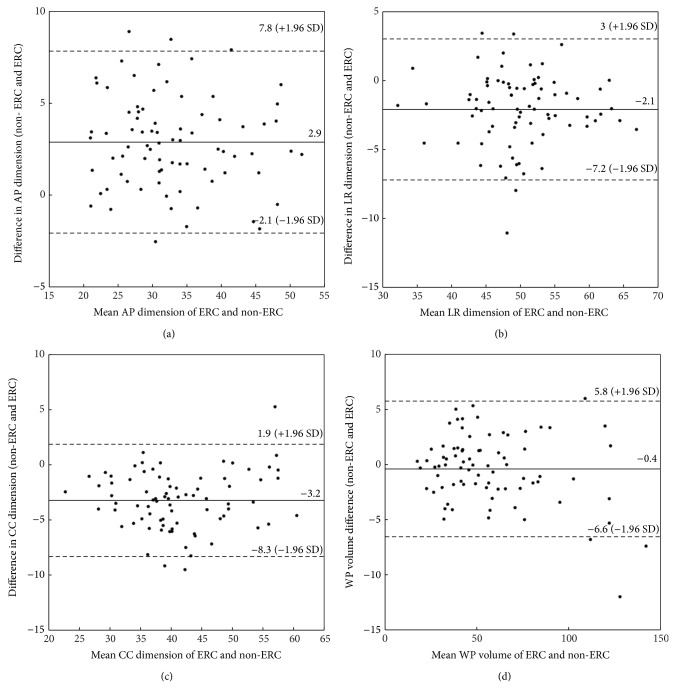
Bland-Altman plots for changes in maximum (a) AP, (b) LR, (c) CC prostate dimensions, and (d) WP volume measured from ERC and non-ERC T2W MRI.

A Bland-Altman plot for WP volume and maximum AP, LR, and CC prostate size measured with non-ERC and ERC MRI is shown in Figure 2.

## 4. Discussion

This study demonstrates that the use of an ERC does not cause significant change in planimetric WP volume estimation on axial T2W MRI. Therefore, use of an ERC should not affect the ability to accurately estimate prostate volumes on MRI. However, significant changes in the maximum AP, LR, and CC prostate dimensions were observed due to the distortion of the prostate shape with the use of ERC. Although the distortion of the prostate has been associated with the use of an ERC, no significant change in WP volume was observed.

In their previous study, Heijmink et al. reported a significant decrease in WP volume (−8.26 ± 3.45 mL) with the use of an ERC [26]. However, we have demonstrated that the WP volume does not significantly change (+0.4 ± 3.1 mL) with the use of an ERC. We suspect that this discrepancy is primarily due to different slice thickness used by Heijmink et al. between their ERC (2.5 mm) and non-ERC MRI (4.0 mm). Additionally, the use of an ERC expands the prostate in the cephalic/caudal direction which further increases the number of apical and basal slices. The change in prostate shape along with a discrepancy in slice thickness will make consistent contouring of the prostate gland between non-ERC and ERC MRI more difficult and hence produce different volume measurements. In this study, a larger population of patients (79 compared to 44 in the previous study) and same slice thickness (3 mm) of T2W MRI for both non-ERC and ERC MRI were used. Furthermore, while contouring the prostate, care was taken to ensure that the same structures were included in the volume measurements by using intraprostatic landmarks. This study is also limited by different in-plane image resolution between non-ERC and ERC MRI. Higher signal-to-noise ratio provided by ERC enables higher in-plane resolution that allows for more accurate delineation of prostate margins for the determination of WP volume. However, no significant difference in WP volume measurement was observed.

These results suggest that volume measurements determined by MR can be relied upon by urologists to choose the most appropriate method of prostate biopsy [13, 31]. PSA density is used as a predictive biomarker and is predicated on an accurate determination of prostate volume. A PSA density lower than 0.15 ng/mL/cm^3^ indicates a reduced likelihood of cancer [10, 11]. In this study, PSA density calculated from either ERC or non-ERC prostate MRI showed that in only 2 out of 79 patients (2.5%), PSA density measurements crossed the 0.15 ng/mL/cm^3^ threshold for active surveillance with the use of ERC. Therefore, PSA density calculated from MRI images from non-ERC and ERC MRI can be reliably used for the clinical practice and evaluation of the prostate. Radiation therapy planning is typically done using CT while MRI is used for the diagnosis purposes [12]. Additionally, these results can potentially improve the registration algorithms developed to integrate CT and ERC/non-ERC MRI to aid the effectiveness of radiation therapy [32, 33].

## 5. Conclusion

This study demonstrates that the use of an ERC in prostate MR imaging at 3T changes the shape of prostate. However, it does not cause a significant change in whole prostate volume measurements by MR planimetry. Furthermore, for therapy planning for prostate cancer and PSA determinations for BPH, the presence of an ERC does not significantly alter WP volumes, which indicates that the prostate is noncompressible in relation to the forces involved during ERC MRI.

## Figures and Tables

**Table 1:** MR imaging parameters used for axial T2W MRI obtained using non-ERC and ERC MRI.

	TR/TE (ms)	FOV (mm)	Matrix	Flip angle (°)	Recon resolution (mm)	Slice thickness (mm)	Slice gap (mm)	Scan time (min)
ERC	5321/120	140	304 × 234	90	0.352	3	0	3.37
Non-ERC	8873/120	180	320 × 217	90	0.273	3	0	7.68

**Table 2:** Whole prostate volume, PSA density, and maximum AP, LR, and CC prostate dimensions measured using T2 weighted MR images acquired with and without ERC.

	ERC	Non-ERC	Difference (ERC-nonERC)	Percentage difference over non-ERC
Whole prostate volume (mL)				
Mean ± std. dev.	57.9 ± 29.2	57.5 ± 28.4	0.4 ± 3.1	0.6 ± 5.7
Median	50.2	50.6	0.1	0.5
Range	17.4–146	17.7–139	−6.0–12.0	−12.2–16.7
PSA density (ng/mL)				
Mean ± std. dev.	0.246 ± 0.583	0.245 ± 0.563	0.001 ± 0.026	−0.2 ± 5.6
Median	0.146	0.143	0.000	−0.3
Range	0.027–5.16	0.028–4.97	−0.075–0.19	−14.3–13.9
Maximum anterior posterior distance (mm)				
Mean ± std. dev.	31.5 ± 8.2	34.4 ± 8.0	−2.9 ± 2.5	−8.6 ± 7.8
Median	30.4	32.4	−2.7	−8.1
Range	18.6–50.6	20.7–52.8	−8.9–2.50	−9.7–21.1
Maximum left to right distance (mm)				
Mean ± std. dev.	51.0 ± 6.8	48.9 ± 6.8	2.1 ± 2.6	4.5 ± 5.8
Median	51.1	48.9	2.0	4.1
Range	33.1–68.6	31.2–65.1	−3.4–11.1	−7.4–26.0
Maximum craniocaudal distance (mm)				
Mean ± std. dev.	42.8 ± 8.3	39.6 ± 8.5	3.2 ± 2.6	8.8 ± 7.0
Median	41.6	37.9	3.1	8.1
Range	23.9–62.8	21.5–59.6	−5.27–9.5	−8.84–26.7
